# Exploring Monitoring Systems Data for Driver Distraction and Drowsiness Research

**DOI:** 10.3390/s20143836

**Published:** 2020-07-09

**Authors:** António Lobo, Sara Ferreira, António Couto

**Affiliations:** Research Centre for Territory, Transports and Environment, Faculty of Engineering of the University of Porto, 4200-465 Porto, Portugal; sara@fe.up.pt (S.F.); fcouto@fe.up.pt (A.C.)

**Keywords:** driver distraction, drowsiness, retrospective data, driver monitoring systems

## Abstract

Driver inattention is a major contributor to road crashes. The emerging of new driver monitoring systems represents an opportunity for researchers to explore new data sources to understand driver inattention, even if the technology was not developed with this purpose in mind. This study is based on retrospective data obtained from two driver monitoring systems to study distraction and drowsiness risk factors. The data includes information about the trips performed by 330 drivers and corresponding distraction and drowsiness alerts emitted by the systems. The drivers’ historical travel data allowed defining two groups with different mobility patterns (short-distance and long-distance drivers) through a cluster analysis. Then, the impacts of the driver’s profile and trip characteristics (e.g., driving time, average speed, and breaking time and frequency) on inattention were analyzed using ordered probit models. The results show that long-distance drivers, typically associated with professionals, are less prone to distraction and drowsiness than short-distance drivers. The driving time increases the probability of inattention, while the breaking frequency is more important to mitigate inattention than the breaking time. Higher average speeds increase the inattention risk, being associated with road facilities featuring a monotonous driving environment.

## 1. Introduction

Driving is a complex task influenced by many circumstances, some of that are directly related to driver behavior and personality and others are more concerned with the driving environment. Almost all the traffic crashes and/or traffic conflicts occur from the conjugation of these two factors: behavior and environment. Driving behavior itself is not exclusively dependent on driver characteristics, such as age, gender, and health, but also on the journey type and motivation [1].

One of the main causes of traffic crashes related with behavioral aspects is driver inattention. Therefore, the phenomenon of inattention has been widely studied, demonstrating that, in general, this type of human-related events is associated with a degraded driving performance, as well as with a significant detriment of the cognitive performance (e.g., the reaction time), leading to a negative impact on road safety [2,3].

Regan et al. [4] defined driver inattention as “insufficient, or no attention, to activities critical for safe driving” and can be divided in different subcategories, such as distraction and drowsiness. This general definition leads to a deep discussion about its key manifestation. For example, the diversion of attention can be originated by any visual, auditory, physical or cognitive stimulus that interfere with the critical activities for safe driving (e.g., looking at other vehicles or at the side mirrors) and by competing activities, which could be anything from daydreaming to the interaction with passengers or digital devices.

Many past studies addressing driver distraction and drowsiness were based on epidemiological data from crash databases [5,6,7], empirical data obtained from test tracks or driving simulators [8,9,10], or naturalistic driving studies (NDSs) [11,12,13,14,15,16,17,18,19,20,21]. NDSs have gained relevance in this field of research in relation to driving simulation, with the argument that cognitive distraction may have a measurable effect in the laboratory, but the actual risks for driving are much lower than in real environment [2,11]. NDSs provide the environmental conditions required to engage drivers to distraction and drowsiness, as well as the type of pre-crash driver behavior data that is necessary to research the relative near crash/crash risk associated to inattention. This method traditionally requires that reliable and unobtrusive instrumented packages be installed on vehicles to monitor the driver, the vehicle, and the surrounding environment. This kind of study is both expensive and time-consuming, and therefore is not easily achievable. However, according to Kuo et al. [22], recent advances in sensing technology have facilitated a broad adoption of NDSs to explore driver behavior as it occurs in the real world.

Motivated by the potential serious consequences of driver inattention, new driver monitoring systems (DMSs) and warning devices have been proposed as standard equipment in some vehicles or as aftermarket devices. Commercial DMSs open up new possibilities for the analysis of real-world driving, allowing to collect big volumes of data for long periods of time and to ease the driver’s feeling that he/she is taking part on an experiment. On the other hand, the lack of control over the technology development and data collection is the main disadvantage of using retrospective data. Nevertheless, the fast development of a technology embraced by all the major players in the automotive industry has resulted in recent years in the presentation of accurate and reliable DMSs. There are several studies describing the development of new methods and technology to improve the accuracy of driver distraction and drowsiness detection, exploring facial image processing [23,24,25,26,27,28], heart rate and brain activity [26,29], and, more recently, dynamic driving parameters and infrastructure information [27,28]. However, few studies have been dedicated to explore DMS data from the perspective of driver inattention and its consequences for road safety [2,30].

In this context of emerging data sources, this study explores retrospective data to analyze driver inattention. Data was gathered by two types of DMS that monitor and alert the driver when he/she gets distracted or drowsy: one type consisted in dedicated devices installed on commercial fleet vehicles, and the other was a smartphone application available to the general public. Both DMSs have been developed by HealthyRoad Biometric Systems (Porto, Portugal), a private company that develops facial biometric technology for the automotive sector. A preliminary data processing on the historical travel records of each driver was conducted using a cluster analysis, with the objective of identifying mobility patterns that could lead to an increase of drowsiness and distraction. Then, a probabilistic regression model was used to find, from the available risk factors, those that have greater impacts on the distraction and drowsiness levels of each trip, determined by the number of alerts emitted by the DMSs. Assuming distraction and drowsiness as safety-critical events, the risk factors are assessed by applying an ordered probit model separately for each type of event. The analyzed risk factors include variables that are directly obtained from the DMS records, such as the driving time and the number and duration of breaks, as well as the mobility patterns found in the cluster analysis.

Given that the aim of this study is to explore DMS data to analyze risk factors contributing to driver inattention, the literature overview presented in the next section is devoted to previous NDSs focused on this topic. The paper proceeds with the presentation of the available data sources, followed by the description of the data preprocessing methods that were required to pursue the objectives of this study. Then, the methodological approach to identify and analyze the risk factors related to driver distraction and drowsiness is detailed. The results of both distraction and drowsiness models are presented and explored in depth in the following section, which includes the definition of different scenarios that allow for a more insightful discussion. Finally, and due to the retrospective nature of the used data, the limitations of the study are debated in the last section, alongside with some closing remarks.

## 2. Related Work

Large scale NDSs have been conducted across the globe with the aim of understanding driver behavior in real-world settings, where the constraints of participant recall of events or ecological validity are removed. Klauer et al. [11], in their 100-Car Naturalistic Driving Study, referred that NDSs promote a more “natural” driving behavior than driving simulators, being preferable for the analysis of the complex phenomena associated with driver inattention. This study estimated that the risk of safety-critical events (SCEs), i.e., crashes and near crashes, is four- to six-times higher for drowsy drivers and three times higher for distracted drivers engaging in secondary tasks in comparison to attentive drivers.

In fact, most studies relating SCEs with distraction report that the driver’s engagement in tasks unrelated to driving is the main risk factor. However, the increase of the inattention risk depends on the visual, manual and/or cognitive demand of each task. Olson, et al. [12] found that 60% percent of SCEs involving professional drivers were associated with the engagement in non-driving related tasks. Additionally, the results suggest that the more complex the task, the higher the SCE risk. The risk increases with the time spent looking away from the road and may be up to 23-times higher for the most demanding of the analyzed tasks (texting messages on the mobile phone) than the baseline conditions. In total, the authors identified 16 different causes of distraction that are statistically significant for the increase of SCE risk. However, Klauer et al. [11] noted that short glances away from the roadway (under 2 s), for instance, to scan the vehicle’s surroundings, do not increase the risk in a relevant way. A large array of distraction causes was also identified by Hanowski et al. [13]. In a study involving 41 truck drivers, the authors recorded 178 SCEs associated with 34 unique types of distraction. Once again, visually-demanding tasks carry the highest degree of risk. Hickman et al. [14] observed from a sample of 207 truck and bus drivers that texting, dialing or reaching a mobile phone increases the risk of SCEs, but talking on the phone does not have a significant effect, corroborating that using a phone while driving should not be considered as a single dichotomous task.

Other studies focused on distraction analyzed the effects of drivers’ personal characteristics. Guo et al. [15] conducted a very large NDS in the US involving 3542 drivers, demonstrating that, although visual-manual distractions are ubiquitous in all age groups, cognitive distraction may have a larger impact on young drivers. Green et al. [16] examined video frames from 96 drivers and concluded that male drivers are slightly more prone to distraction than female drivers; male drivers were associated with 56% of the distraction events, defined by four or more successive frames in which the driver was not looking at the forward scene.

The research on driver drowsiness using NDSs has been mainly devoted to the analysis of sleeping/resting patterns and, to a lesser extent, of driver and trip characteristics. Chen et al. [17] identified four distinct sleep patterns among 96 truck drivers based on sleep duration, sleep start/end point in a non-working period, and the percentage of sleeping time in relation to the duration of the non-working period. A negative binomial regression was used to evaluate the association between the sleep patterns and SCE rate, adjusted for driver demographic information. The results showed that the sleep pattern with the highest risk was associated with shorter sleeps, sleeping in the early stage of a non-working period, and less sleep between 1 a.m. and 5 a.m. Male drivers with less professional driving experience and higher body mass index were also associated with increased driving risk. Sparrow et al. [18] used data from smart wristbands, psychomotor vigilance tests and subjective sleepiness rated through the Karolinska Sleepiness Scale (KSS) to assess the effects of sleep patterns on the driving performance of 106 professional drivers. Using a mixed-effects ANOVA approach, the authors concluded that non-working periods covering at least two nights help to mitigate drivers’ fatigue, providing a greater opportunity for sleep recuperation. Pylkkönen et al. [19] also used wearable monitoring devices data and KSS ratings from 54 truck drivers to explore the relations between insufficient daily sleep (under six hours) and severe sleepiness (KSS ≥ 7) and the type of driving shift. Using generalized estimation equations, it was found that severe sleepiness is six to nine times more likely to occur on first night shifts compared to day/evening shifts, and that insufficient daily sleep is three-times more likely in consecutive night shifts than in day/evening shifts. Drivers are also less likely to use sleepiness countermeasures, such as ingesting caffeine or taking a nap, during statutory breaks than during voluntary breaks.

The relation between driving time and SCEs was analyzed by Hanowski et al. [20], who reported a peak in the rate of incidents during the first driving hour from a sample of 98 professional drivers. The results showed no statistical differences between the following driving hours in 11-h shifts. However, a subsequent study by Soccolich et al. [21] involving 97 professional drivers showed a positive correlation between driving hours and SCEs using a negative binomial model. Follow-up analyses revealed that the SCE rate in the 11th driving hour is significantly higher only when compared to the first and second driving hours, and that the risk of being involved in an SCE increases with the accumulated work hours, consisting of both driving and non-driving hours. Breaks were found to counteract the negative effects of time-on-task, whether the break consisted of work or restful activities. This effect is particularly relevant in the first driving hour after a break, in which the reduction of the SCE rate may be up to 50% in relation to the last driving hour before a break.

The literature shows that NDSs that consider exposure variables, such as driving and resting times, to analyze driver inattention are particularly scarce, especially in relation to driver distraction. Moreover, NDSs focused on driver drowsiness are usually devoted to professional drivers. These issues further stress the relevance of the present study and of the use of DMS data for driver inattention research on comprehensive driver samples.

## 3. Data

### 3.1. Data Sources

This study uses data collected by two types of DMS: an embedded system installed onboard test vehicles, consisting of a fixed dashboard infrared camera to obtain images of the driver’s face and eyes, and a smartphone camera-based application that was available at no cost on Google Play Store. These DMSs use deep learning algorithms to process facial images and detect distraction and drowsiness events, warning the driver for dangerous behavior through an audible alert (similar for both types of event). To classify a distraction event, the systems uses a combination of head position and eye gaze monitoring data, producing an alert each time the driver is detected taking his/her eyes off the road for more than two seconds. The systems automatically calibrate head yaw and pitch before each usage by asking the driver to stare at the road for a few seconds. Additionally, the systems can distinguish between six gaze focus areas: road, dashboard, central console, rearview mirror, left-side mirror, and right-side mirror. To detect a drowsiness event, the systems track eye blinking, emitting an alert when the driver closes his/her eyes for more than one second. The smartphone application featured a tutorial video about how to position the smartphone on the vehicle’s dashboard and a warning signal to alert the driver when the camera was wrongly positioned and unable to detect his/her face.

Both DMSs were, at the time of the experiments that originated the data for this study, in a prototype stage. Previously to these experiments, the DMS developer had conducted road tests with simultaneous video recording to assess the accuracy of the classification algorithms, considering different individuals and driving conditions, including road environment (urban, rural, and highway), lighting (different hours and weather conditions), camera positioning, and the use of eyeglasses. The videos were observed and the alerts were verified using predefined heuristics. The developer claims an average accuracy of head position and eye gaze classification according to the focus area of 93% and 92%, respectively. The developer also reports an average recall and precision of 93% for the classification of eye closures.

The DMSs were developed with the objective of warning drivers about inattentive behavior rather than providing data for road safety research. Therefore, the data that supports this study may be classified as retrospective data, as the authors did neither supervise the development and implementation of the DMSs nor the data collection and management. The access to the driving records was granted after these processes and after the removal of the drivers’ personal identifiers. The authors of this study did not have access to any video recordings.

The provided data contains the classification of alerts as distraction or drowsiness events, but do not distinguish between types of distraction (e.g., texting or looking outside the vehicle) or between types of drowsiness (e.g., microsleeps or fatigue). The embedded DMS was installed on commercial fleet vehicles, predominantly used by professional drivers. The smartphone-based DMS was available to the general public, thus there is no a priori information about the type of driver that used the system. In both cases, there is also no information about the drivers’ personal characteristics. The variables recorded by the two systems are the timestamp and GPS position at the beginning and the end of each continuous driving activity, the timestamp, GPS position, instant vehicle speed and type of alert each time a distraction or drowsiness warning is emitted, and unique identifiers for the driver (driver ID) and the driving activity (sub-journey ID). A continuous driving activity, hereinafter referred to as sub-journey, is a trip between two points that is truncated by the system according to criteria predefined by the developer, based on the vehicle’s displacement and stopping time. Basically, sub-journeys aim to represent trips from when the driver starts the vehicle until he/she turns it off. In this sense, each sub-journey includes brief stops caused by traffic conditions and yielding, but does not include resting periods.

### 3.2. Data Cleaning

The database provided for this study consists in the records of sub-journeys and alerts obtained between December 2015 and September 2016 by both types of DMS described above. To account for the effects of the resting time between sub-journeys on the risk of inattention, the concept of journey was established by grouping sequential sub-journeys performed by the same driver. Each journey was truncated when the breaking time between successive sub-journeys surpassed nine hours. The nine-hour threshold is based on the Regulation (EC) No 561/2006 from the European Parliament and of the Council of 15 March 2006 that establishes a minimum daily rest period of nine hours for road transport [31]. Following the definition of journeys, three data cleaning processes were performed. First, successive alerts separated by less than five seconds were considered as a single event. Second, it was noted that the first sub-journeys of some journeys were very short and presented an abnormal number of alerts, mainly related to distraction and the use of the smartphone application. These sub-journeys are probably trials conducted by first-time users, which are not representative of inattention patterns. To exclude these sub-journeys from the analysis, first sub-journeys with a driving time smaller than 10 min were not considered. Finally, it was observed that some sub-journeys had a combination of travel distance and travel time that correspond to very low or very high average speeds. Very low speeds may be attributed to small movements that are not meaningful for the purpose of this study (e.g., moving the vehicle from one parking spot to another inside a logistic center). Very high speeds are associated with data collection errors, specifically related to the GPS position. Therefore, only the sub-journeys with an average travel speed between 10 and 150 km/h were analyzed.

In the end, 11,034 distraction events, 6949 drowsiness events, 1658 sub-journeys, and 330 drivers observed with both DMSs were considered in this study. A brief observation of the data collected separately by each DMS allows knowing beforehand that there are much more occasional users of the smartphone application than of the embedded DMS, with the former system having more single-time users and a lower ratio of sub-journeys per driver ID. This fact denotes that the predominant users of the smartphone application may have different characteristics from the users of the embedded DMS, which is admittedly associated with professional drivers. Therefore, the records from both DMSs were merged into a single database that aims to cover a more comprehensive range of users and that allows exploring different driver profiles.

### 3.3. Data Preprocessing

After the data cleaning, new variables were derived from the DMS records to address the objectives of this study. Because drivers’ personal characteristics are not available, drivers were characterized according to their profile based on historical travel data. Therefore, groups of drivers with different mobility patterns were defined through a cluster analysis. For that, a database was built by aggregating for each driver the information collected by the DMSs using the corresponding driver ID. For each driver, the following variables were determined: total traveled distance (*TotTravelDistance*), average journey distance (*AvgJourneyDistance*), average sub-journey distance (*AvgSubJourneyDistance*), average number of sub-journeys per journey (*SubJourneysPerJourney*), average speed (*AvgSpeed*), average breaking time before sub-journeys (*AvgBreakTime*), and average breaking frequency (*AvgBreakFrequency*). The average speed was computed for each driver based on the total traveled distance and driving time, excluding breaks. The average breaking frequency was defined as the total number of breaks performed by each driver divided by the total driving time.

The clustering to define drivers’ mobility patterns was performed using the k-means algorithm. This algorithm requires to define *a priori* the number of clusters *k*, thus it was run iteratively, starting with *k* = 2, using the data mining software *RapidMiner* (RapidMiner, Inc., Boston, MA, USA). The results for two clusters are easily interpretable and clearly identify two distinct driving patterns: short-distance and long-distance drivers. For *k* ≥ 3, the clustering started to result in over-segmentation, isolating small groups of drivers that could not be considered as representative of general behavior patterns. Therefore, the solution with two clusters was selected, being presented in Table 1.

Cluster 1 represents short-distance drivers that performed short journeys with very few interruptions. Hence, the journeys of the average driver in this cluster are composed by a single and short sub-journey with no breaks. Around 90% of the drivers in Cluster 1 used the smartphone application, probably in a sporadic way, accumulating much smaller distances than the drivers in Cluster 2. This cluster represents the frequent travelers that have accumulated greater travel distances and performed longer journeys. The number of sub-journeys per journey, the breaking time and the breaking frequency are higher in Cluster 2, with the drivers in this cluster being responsible for approximately 68% of the analyzed sub-journeys, despite being much less than the drivers in Cluster 1. The average speed of drivers in Cluster 2 is higher, denoting that the use of high-speed road facilities is more likely on longer journeys.

After the definition of the drivers’ mobility patterns, the risk factors associated with driver inattention based on the available retrospective data were analyzed using a probabilistic regression approach. For that, the information collected by the DMSs was aggregated for each sub-journey in a second dataset, using the corresponding sub-journey ID. Because each sub-journey, as defined above, was performed by a single driver, two binary variables (*ShortDistanceDriver* and *LongDistanceDriver*) were used to set the type of driver that made each sub-journey, according to the clustering results. Additionally, the following trip characteristics were computed: sub-journey time duration (*SubJourneyTime*), accumulated driving time at the end of the current sub-journey (*AcDriveTime*), breaking frequency (*BreakFrequency*), time spent in the last break (*BreakTime*), percentage of breaking time (*PercBreakTime*), average sub-journey speed (*AvgSubJourneySpeed*), and a binary variable for first sub-journeys (*FirstSubJourney*). The accumulated driving time is the sum of the time duration of the current and the previous sub-journeys in the same journey. Conversely, the percentage of break time is given by the total time spent in breaks prior to the current sub-journey divided by the accumulated journey time, including breaks. The break frequency is the number of breaks divided by the accumulated driving time. Because there is no break before the first sub-journey of each journey, the break time of the first sub-journeys is equal to zero. The break time aims to be a proxy for the drivers’ resting level before engaging in a new driving activity, but this level is unknown for the first sub-journey. In this sense, a binary variable for first sub-journeys was created to capture the discontinuity of the break time effect in the vicinity of zero, allowing differentiating the first sub-journeys (unknown resting level) from the following ones with very small break/rest times. In addition to driver and trip characteristics, a binary variable for the use of the smartphone application (*SmartphoneApp*) was considered to capture the effects of using a different hardware in relation to the embedded DMS. Although the algorithms between both DMSs are identical, the smartphone application implies less control over the quality of the facial images, which depends on the camera resolution, and over the camera positioning. Table 2 contains the general data description of the variables used in the probabilistic regression modeling.

From the 1668 sub-journeys considered in the probabilistic regression modelling, 1178 (70%) were retrieved from the embedded DMS and 480 (30%) were obtained with the smartphone application. Regarding the data collected with the embedded DMS, around 90% of the sub-journeys are associated with long-distance drivers (1062 observations), while 10% are associated with short-distance drivers (116 observations). In relation to the smartphone application, short-distance drivers account for approximately 86% of the recorded sub-journeys (415 observations) and long-distance drivers represent the remaining 14% (65 observations).

## 4. Methodological Approach

The number of alerts per journey may be derived into a scale representing an increasing risk of driver inattention, thus being suitable to the application of ordered choice models [32]. Therefore, both the number of distraction alerts and the number of drowsiness alerts per sub-journey were converted into the following categories: 0 (no alerts), 1 (one alert), 2 (two alerts), 3, (three alerts), and 4 (four or more alerts). In this study, a backward stepwise ordered probit model (OPM) was applied separately for each type of inattention event. The discrete distraction or drowsiness risk levels ($y_{i}$) are mapped to an underlying continuous latent variable ($y_{i}^{*}$), as specified by:

(1) $$y_{i}^{*}=x_{i}\beta +\varepsilon_{i},$$

where $y_{i}^{*}$ is the continuous latent variable, *x_i_* is the vector of exogenous variables, *β* is the vector of parameters to be estimated, *ε_i_* is the random disturbance term, and *i* is the observation in the sample (*i* = 1, …, *n*). The unobserved component $y_{i}^{*}$ is associated with the inattention risk factors. The link between this latent variable and a given observed number of alerts (*y_i_*) can be defined by:

(2) $$y_{i}=\left\{ \begin{array}{l} 0\;if\;y_{i}^{*}\leq 0\\ 1\;if\;0\;<\;y_{i}^{*}\leq \mu_{1}\\ 2\;if\;\mu_{1}<\;y_{i}^{*}\leq \mu_{2}\\ 3\;if\;\mu_{2}<\;y_{i}^{*}\leq \mu_{3}\\ 4\;if\;y_{i}^{*}>\mu_{3} \end{array}\right.$$

where *μ*_1_, *μ*_2_, and *μ*_3_ are thresholds for the inattention levels to be estimated. The resulting probability expressions for an individual *i* are represented by:

(3) $$\mathrm{Prob}\left(y_{i}=0|x_{i}\right)=\Phi \left(-x_{i}\beta \right)$$

(4) $$\mathrm{Prob}\left(y_{i}=1|x_{i}\right)=\Phi \left(\mu_{1}-x_{i}\beta \right)-\;\Phi \left(-x_{i}\beta \right)$$

(5) $$\mathrm{Prob}\left(y_{i}=2|x_{i}\right)=\Phi \left(\mu_{2}-x_{i}\beta \right)-\;\Phi \left(\mu_{1}-x_{i}\beta \right)$$

(6) $$\mathrm{Prob}\left(y_{i}=3|x_{i}\right)=\Phi \left(\mu_{3}-x_{i}\beta \right)-\;\Phi \left(\mu_{2}-x_{i}\beta \right)$$

(7) $$\mathrm{Prob}\left(y_{i}=4|x_{i}\right)=1-\;\Phi \left(\mu_{3}-x_{i}\beta \right),$$

where Φ(.) is the cumulative function of the normal distribution assumed for the error term *ε*.

The OPM was applied in different steps, starting from the complete set of independent variables and removing the least significant in each step until all the retained variables are statistically significant at 5% level. Model estimations were performed through a simulated maximum likelihood method developed by Greene [33], using the econometric software Limdep 9.0 (Econometric Software, Inc., Plainview, NY, USA).

## 5. Results and Discussion

The parameter estimations of the OPM for driver distraction and drowsiness are presented in Table 3.

The results reveal that long-distance drivers tend to be less prone to distraction and drowsiness than short-distance drivers. As expected, a greater sub-journey time increases the probability of one or more alerts being emitted by the DMS, which means that increasing the driving time without taking a break increases both distraction and drowsiness levels. Continuous driving has been associated with increased inattention risk by other authors, particularly in the case of drowsiness. For instance, a survey-based study by Mahajan et al. [34] found a statistically significant difference between driving continuously up to one hour and up to four hours. Van der Hulst et al. [35] noted that fatigue, which was associated with the deterioration of perceptual-motor performance, increased over time during a driving simulator study. Wang et al. [36] found that the driver’s active fatigue level, associated with the mean heart rate, increased with driving time during a field operational test focused on the effects of the roadside landscape. In turn, the accumulated driving time during a journey was removed from both models due to the lack of statistical significance. In fact, the existing literature reports some contradictory effects associated with this variable; while Hanowski et al. [20] observed a peak of SCEs in the first hour of 11-h driving shifts performed by professional drivers, Soccolich et al. [21] noted that incidents increase with driving time, but no significant differences were found from the eighth through the 11th hour.

The breaking frequency has a positive effect to lower the number of distraction and drowsiness alerts. The time spent in the last break is not statically significant in both models. The percentage of breaking time during the journey does not also have a statistically significant effect on drowsiness, but it is associated with an increase of the probability of distraction, suggesting that highly unbalanced journeys with long resting times may defocus the driver’s attention from the driving task. Therefore, it is possible to conclude that the number of breaks made during a journey is more relevant to the mitigation of distraction and drowsiness than the time spent on these breaks. In accordance, the number of breaks and the duration of the last sub-journey have an impact on driver inattention, while the total journey duration is not relevant. The positive effects of breaking frequency were reported by Soccolich et al. [21], who referred that taking a break significantly reduces the SCE rate in the first driving hour after that break, regardless if the break is dedicated to work or to take a rest. Jung et al. [7] and Bunn et al. [37] analyzed official crash databases to find a negative correlation between road crashes caused by drowsy driving and the proximity to rest areas.

Regarding the effects of speed, it was found that the average sub-journey speed is positively correlated with the level of distraction and drowsiness. Usually, higher average speeds are associated with rural/monotonous road facilities, which could explain the higher probability of drowsiness. In some cases, long motorway sub-journeys could also imply the inevitability of drivers’ engagement in secondary tasks, increasing the risk of distraction. In fact, a field operational test conducted by Iseland et al. [38] allowed to conclude that long-distance truck drivers use secondary tasks to alleviate boredom and drowsiness, and for social interaction. These secondary tasks are described as ‘‘necessities” (e.g., reaching food, eating, drinking, removing a jacket, rubbing their face, and adjusting the seat), interactions with technology (e.g., mobile phone and infotainment systems), or administrative tasks.

Finally, the impacts of a sub-journey being the first one in a journey are contradictory between the two models. The probability of drowsiness events in the first sub-journey is intuitively smaller; in Sparrow et al. [18], a longer off-duty period preceding a work journey is associated with decreasing levels of drowsiness. However, the first sub-journey reveals an increased probability of distraction. This could be explained by the fact that the driving task requires an initial short period of adaptation to focus on the driving process. As previously mentioned, Hanowski et al. [20] observed a higher number of incidents involving professional drivers during the first hour of 11-h driving shifts, but, it should be noted that this work does not distinguish between incident causes.

The binary variable for the use of the smartphone application is not statistically significant in the distraction model, denoting that both DMSs perform in a similar way to detect distraction. The variable was removed during the backward stepwise procedure, with the effects of the other variables remaining stable after the removal. In relation to the drowsiness detection, the statistical significance of the use of the smartphone application suggests that there are some differences between both DMSs. As previously noted, since the classification algorithms are similar, this variable is capturing uncontrolled events related to the system’s use, such as the quality of the camera and the positioning of the smartphone during the experiments. The negative coefficient may suggest some underreporting of drowsiness events by the smartphone application in relation to the embedded DMS. Given these results, the simultaneous consideration of the DMS type and the driver profile does not introduce collinearity issues in the developed models.

In addition to the estimation of the models’ parameters, the elasticities at the sample mean of the continuous variables in relation to the probability of no alerts being emitted (*y_i_* = 0) were determined from the corresponding marginal effects. These elasticities, presented in Table 4, allow for a better understanding of the contribution of each variable to the occurrence of one or more distraction or drowsiness events.

The sub-journey time has elasticities at the sample mean in relation to Prob(*y_i_* = 0) of −0.226 and −0.201 for distraction and drowsiness, respectively. Therefore, an increase of 10% in the sub-journey time implies an increase of around 2.3% and 2.0% in the probability of having one or more distraction and drowsiness events on that sub-journey, respectively, while maintaining constant the remaining variables. In relation to the breaking frequency, an increase by 10% of the number of breaks during a journey, ceteris paribus, increases the probability of no alerts being emitted alerts during the sub-journey by approximately 1.1% for distraction and 1.3% for drowsiness. Looking at the percentage of breaking time per journey, the elasticity at the sample mean indicates that an increase of this variable by 10% produces an increase of 1.7% on the probability of at least one distraction event occurring in that sub-journey, ceteris paribus. The elasticity at the sample mean of the average sub-journey speed shows that a 10% increase in this variable, and consequently in the sub-journey distance, since the remaining variables in the model are kept constant, leads to a decrease of 4.1% in the probability of no distraction alerts being emitted. In the same situation, the decrease of the probability of zero alerts for drowsiness is significantly smaller (1.2%).

Because the elasticity analysis for each model and category of the dependent variable would be quite extensive and repetitive, the response of both models in terms of the probabilities associated with each category is analyzed for the following scenarios:

- S1 (baseline scenario): a long-distance driver performs a one-hour sub-journey at an average speed of 80 km/h, accumulating four driving hours and two breaks during the same journey and spending 15% of the journey time in breaks.
- S2: similar to S1, but performed by a driver that usually travels short distances;
- S3: similar to S1, except that the last sub-journey is performed at half of the speed (40 km/h) and takes the double of time (two hours);
- S4: similar to S1, except that the driver makes twice the stops (four breaks).

The probabilities associated with each level of inattention were computed using Equations (3)–(7). The results are shown in Table 5.

In general, the probabilities associated with each scenario decrease from the category without alerts to the category with three alerts, increasing for the category with four or more alerts. In the cases of S2 for distraction and S3 for drowsiness, the probability of occurrence of four or more alerts during the last sub-journey surpasses the probability of zero alerts.

Comparing S2 with the baseline scenario (S1), and according to the previous results presented in Table 3, it is possible to observe that short-distance drivers are much more prone to distraction than long-distance drivers, with short-distance drivers having more than twice the probability of incurring in a high number of distraction events during long sub-journeys than long-distance drivers (38% *versus* 16%). Long-distance drivers also perform better with respect to drowsiness, with a probability of zero alerts of 46% (42% for short-distance drivers). Considering that 70% of the individuals included in the long-distance drivers’ cluster are associated with the use of the embedded DMS installed on commercial vehicles, this cluster is probably composed by a higher percentage of professional drivers than the cluster of short-distance drivers. In this sense, professional drivers may be more experienced in adopting strategies to mitigate inattention.

In relation to S3, it is possible to observe that spending a greater time to travel the same sub-journey, and consequently lowering the average speed, has virtually no effects on distraction. In turn, the probability of not experiencing drowsiness decreases from 46% in S1 to 32% in S3, and the probability of incurring in a high number of events (four events or more) rises from 25% in S1 to 38% in S3.

Finally, the increase of breaking frequency assumed in S4, in relation to S1, has a slightly higher potential to reduce drowsiness than to reduce distraction. The probability of zero drowsiness alerts during the sub-journey increases from 46 to 53% (57 to 60% for distraction), while the probability of occurrence of four or more drowsiness alerts decrease from 25 to 20% (16 to 14% for distraction).

Comparatively to S1, both S3 and S4 produce a greater impact on drowsiness than on distraction. This fact denotes that drowsiness, as a physiological state, is much more sensitive to the continuous driving time and breaking frequency, as these factors are directly related to the level of tiredness experienced by the driver. On the other hand, distraction events may be triggered by a countless number of stimuli from inside and outside the vehicle, which contributes to diminish the effects associated with the driver’s experience and with the journey and driver’s characteristics.

## 6. Limitations and Closing Remarks

The exploration of retrospective data has clear advantages, particularly reflected by an easy access to information for a wide range of applications. However, this sampling process has some limitations. The most relevant concern lies on the fact that retrospective data can have multiple sources and motivations, usually far from the objectives of scientific research, resulting in the availability of uncontrolled information for this purpose.

Because of confidentiality issues, it was not possible to have access to all the information about the experiments carried out to obtain the data used in the research, including the distraction and drowsiness classification algorithms, a full characterization of their calibration and validation processes, and video recordings. The used DMSs also do not provide detailed information about the origin of the distraction events, which would be a limitation if the goal was to correlate specific types of distraction and driver’s performance. However, notwithstanding the causes of distraction, the main effect of this type of inattention remains, as safe driving is compromised by the vulnerability to risky situations.

In addition, variables such as the trip motivation, the driver’s personal characteristics (e.g., gender, age and occupation), and some complementary real-time data about the driver’s physiological state and the characteristics of the road environment are examples of missing information that could have improved the analysis and provided a better understanding of the results. For instance, as noted by Fitzharris et al. [3] in a study using similar technology, no one can fully ensure that the DMS records of a single journey are always associated to the same driver.

To address the limitations regarding the lack of information about the drivers’ characteristics, a driver profiling was made to define different profiles based on travel behavior, which was enabled by the use of a rich dataset built from the observations of the two types of DMS. However, using records from two different systems requires caution to keep the validity of the results. The developer claims high average levels of accuracy for their classification algorithms, but despite the fact that these algorithms are similar in the embedded system and the smartphone application, it is not possible to fully guarantee that the accuracy of the smartphone application is not affected by the diversity of characteristics of the users’ smartphones (e.g., camera resolution). Therefore, a binary variable to set the type of DMS was considered in both models to capture such effect, which apparently only affects the drowsiness classification.

Despite the mentioned limitations, studies based on retrospective driving data can complement the results of other studies and provide useful knowledge about the risk factors contributing to inattention, particularly by addressing the aspects related to exposure, such as driving and breaking times, and by achieving large samples of drivers from different groups without requiring the resources of purposely designed NDSs. The results obtained in this study indicate that the main risk factor associated with driver inattention is the continuous, non-stopping, driving time. The duration of breaks and the total time spent in the journey are not relevant. These results are in line with those from previous research based on different data collection methods, including naturalistic driving, driving simulation, questionnaires, and crash databases. However, it should be noted that the obtained results are not fully comparable to other studies in which no inattention feedback was provided to the driver, particularly regarding the magnitude of the effects. In fact, when feedback is given to the driver, in this case in the form of an audible alert, the driver can take countermeasures to mitigate inattention (e.g., take a rest or have a coffee), altering the journey in relation to the situation in which there is no feedback [3].

From the obtained results, it is also possible to observe that drivers that perform mostly small journeys, i.e., typically non-professional drivers, are particularly exposed to risk when they have to travel longer distances. In contrast to professional drivers, non-professional drivers are not subject to any kind of regulations about driving times and resting periods. However, the differences between professional and non-professional drivers should be confirmed in future studies, particularly by considering drivers’ personal characteristics and personality traits.

## Figures and Tables

**Table 1:** Cluster centroids.

Variable	Cluster 1	Cluster 2
TotTravelDistance (km)	44.36	1873.75
AvgJourneyDistance (km)	26.28	161.73
AvgSubJourneyDistance (km)	24.29	75.89
SubJourneysPerJourney	1.08	3.09
AvgSpeed (km/h)	52.22	81.15
AvgBreakTime (h)	0.02	0.87
AvgBreakFrequency (no./h)	0.11	1.67
Number of drivers	295	35

**Table 2:** Data description.

Variable	Mean	Standard Deviation	25th Percentile	75th Percentile	Relative Frequency (%)
SubJourneyTime (h)	0.76	0.94	0.23	0.82	-
AcDriveTime (h)	2.77	4.67	0.37	3.04	-
BreakFrequency (No./h)	0.90	1.52	0.00	1.20	-
BreakTime (h)	0.62	1.43	0.00	0.42	-
PercBreakTime (%)	22.80	28.84	0.00	40.82	-
AvgSubJourneySpeed (km/h)	54.12	31.32	28.56	74.87	-
FirstSubJourney	-	-	-	-	45.96
ShortDistanceDriver	-	-	-	-	32.03
LongDistanceDriver	-	-	-	-	67.97
SmartphoneApp	-	-	-	-	28.95

**Table 3:** OPM estimations.

Variable	Distraction OPM ^a^		Drowsiness OPM ^b^	
	Coefficient	*p*-Value	Coefficient	*p*-Value
ShortDistanceDriver	−0.822	0.000	−0.630	0.000
LongDistanceDriver	−1.515	0.000	−0.734	0.000
SubJourneyTime (h)	0.422	0.000	0.584	0.000
BreakFrequency (No./h)	−0.174	0.000	−0.319	0.000
PercBreakTime (%)	0.011	0.000	-	-
AvgSubJourneySpeed (km/h)	0.011	0.000	0.005	0.000
FirstSubJourney	0.344	0.001	−0.367	0.000
SmartphoneApp	-	-	−0.584	0.000
μ1	0.427	0.000	0.408	0.000
μ2	0.674	0.000	0.640	0.000
μ3	0.824	0.000	0.777	0.000

^a^ Number of observations = 1658; Log likelihood = −1822; McFadden Pseudo-R^2^: 0.106; AIC = 3664. ^b^ Number of observations = 1658; Log likelihood = −1312; McFadden Pseudo-R2: 0.182; AIC = 2645.

**Table 4:** Marginal effects and elasticities for Prob(*y_i_* = 0).

Variable	Distraction OPM			Drowsiness OPM		
	Marginal Effect	Elasticity	*p*-Value	Marginal Effect	Elasticity	*p*-Value
SubJourneyTime (h)	−0.167	−0.226	0.000	−0.193	−0.201	0.000
BreakFrequency (No./h)	0.069	0.111	0.000	0.106	0.130	0.000
PercBreakTime (%)	−0.004	−0.172	0.000	-	-	-
AvgSubJourneySpeed (km/h)	−0.004	−0.408	0.000	−0.002	−0.123	0.000

**Table 5:** Probabilities of the outcomes of the OPM for different scenarios.

Scenario	Prob(*y_i_* = 0)	Prob(*y_i_* = 1)	Prob(*y_i_* = 2)	Prob(*y_i_* = 3)	Prob(*y_i_* = 4)
**Distraction**					
S1	57	16	8	4	16
S2	30	16	10	6	38
S3	57	16	8	4	16
S4	60	15	7	4	14
**Drowsiness**					
S1	46	16	8	5	25
S2	42	16	9	5	28
S3	32	16	9	5	38
S4	53	16	8	4	20
